# Hybrid Seed Set in Relation with Male Floral Traits, Estimation of Heterosis and Combining Abilities for Yield and Its Components in Wheat (*Triticum aestivum* L.)

**DOI:** 10.3390/plants11040508

**Published:** 2022-02-14

**Authors:** Samira El Hanafi, Souad Cherkaoui, Zakaria Kehel, Miguel Sanchez-Garcia, Jean-Benoit Sarazin, Stephen Baenziger, Wuletaw Tadesse

**Affiliations:** 1 International Center for Agricultural Research in the Dry Areas, Rue Hafiane Cherkaoui, Rabat-Institutes, Rabat B.P. 6299, Morocco; z.kehel@cgiar.org (Z.K.); m.sanchez-garcia@cgiar.org (M.S.-G.); w.tadesse@cgiar.org (W.T.); 2 Physiology Plant Biotechnology Unit, Bio-Bio Center, Faculty of Sciences, Mohammed V University of Rabat, 4 Avenue Ibn Battouta, Rabat B.P. 1014, Morocco; s-cherkaoui@um5r.ac.ma; 3 Asur Plant Breeding, Estrées-Saint-Denis, 60190 Picardy, France; jean-benoit.sarazin@asur-plantbreeding.com; 4 Department of Agronomy and Horticulture, University of Nebraska-Lincoln, Lincoln, NE 68588, USA; pstephen.baenziger@gmail.com

**Keywords:** CHA, floral traits, heterosis, hybrid wheat, GCA, SCA, seed set

## Abstract

Breeding hybrids with maximum heterosis requires efficient cross-pollination and an improved male sterility system. Renewed efforts have been made to dissect the phenotypic variation and genetic basis of hybrid floral traits, although the potential of tailoring the appropriate flower design on seed setting is less known. To this end, elite wheat genotypes were crossed using a chemical hybridizing agent at different doses. A total of 23 hybrids were developed from a partial diallel design; and planted in an alpha lattice design with their parents at two locations in Morocco, for two years, to evaluate for yield components, heterosis and combining abilities. The 13.5 L ha^−1^ dose induced a maximum level of sterility (95%) and seed set showed large phenotypic variation and high heritability. In parallel, seed set showed tight correlation with pollen mass (0.97), visual anther extrusion (0.94) and pollen shedding (0.91) (*p* < 0.001), allowing direct selection of the associated traits. Using the combined data, mid-parent heterosis ranges were −7.64–14.55% for biomass (BM), −8.34–12.51% for thousand kernel weight (TKW) and −5.29–26.65% for grain yield (YLD); while best-parent heterosis showed ranges of −11.18–7.20%, −11.35–11.26% and −8.27–24.04% for BM, TKW and YLD, respectively. The magnitude of general combining ability (GCA) variance was greater than the specific combining ability (SCA) variance suggesting a greater additive gene action for BM, TKW and YLD. The favorable GCA estimates showed a simple method to predict additive effects contributing to high heterosis and thus could be an effective approach for the selection of promising parents in early generations.

## 1. Introduction

As of 2020, the world population was over 7.8 billion and projected to increase by more than 25% to reach 9.9 billion by 2050 [1]. This ever-growing population has led to concerns about the increased demand for food. In order to achieve sustainable agricultural development for food security, it is important to breed resilient high-yielding crops that are suited to suboptimal conditions of growth. Hybrid wheat represents a promising approach that improves yield potential, yield stability across diverse environments and consequently increases global wheat productivity [2]. However, hybrid wheat occupies nearly 1% of the total world wheat area and is produced mainly in Europe, China and India [3]. Central Europe, particularly France, Hungary, and Germany, contains about half of the world’s hybrid-wheat production area and is home to the two leading hybrid seed producer companies ASUR Plant Breeding SAS, (previously SAATEN UNION) and Nordsaat Saatzuchtgesellschaft mbH [4]. All hybrids that are registered in Europe are currently produced by the application of chemical hybridization agents (CHAs), most commonly Croisor^®^ 100 (Sintofen; former Dupont–Hybrinova, Saaten–Union Recherche, France). In China, photoperiodic sensitivity and cytoplasmic male sterility (CMS) systems are being successfully used for grain production [4,5]. Wheat hybrids in India are developed using cytoplasmic male sterility (CMS) systems derived from *Triticum timopheevii* and a CHA approach [2,6].

For the long term success of hybrid breeding programs, adequate exploitation of heterosis, cost-effective hybridization systems, high seed density and the development of high-yielding heterotic groups and patterns must be established [4,7,8,9]. Heterosis, or hybrid vigor, is the superiority of a hybrid in its performance over its corresponding parental inbred lines (mid- or best- parent heterosis). Recently, there has been significant focus on developing successful hybrids through different approaches, for more cost-effective seed production [10,11,12]. Studies based on experimental data in hybrid wheat breeding have demonstrated that best-parent heterosis ranged from −70.4 to 54.3% and −26.9 to 29.2% in two successive years, respectively, using CHA Croisor^®^ 100 on elite winter wheat lines from the University of Nebraska–Lincoln (UNL) and Texas A and M University breeding programs; while mid-parent heterosis for grain yield varied from −15.33% to 14.13% of 112 hybrids produced using a CMS system [8,13].

Despite the hybrid yield advantage, development of practical hybrid seed production systems has not yet reached large-scale hybrid wheat production. This can, in part, be attributed to the high cost of hybrid wheat seed. Therefore, maximizing seed set per spike is an effective approach to tremendously improve the cost-efficiency of hybrid seed production in self-pollinating crops, such as wheat, irrespective of the hybridization system. Floral biology plays an important role in enhancing outcrossing ability. Knowledge of the genetic basis of important floral traits and exploring the genetic variability of parental pairs might offer new perspectives on methods to restore the fertility control system [5,9].

Numerous studies have addressed the importance of appropriate floral and flowering traits as key determinants for potential hybrid parents. Traits like anther extrusion, visual anther extrusion, pollen mass, degree and duration of flower opening, stigma receptivity, anther length, number and longevity of pollen grains show large genotypic variation and moderate to high heritabilities and thus might be integrated for a successful fertility control system [14,15,16,17,18,19,20]. Considerable progress has been made in identifying proxies suitable for allowing direct and indirect selection of associated hybrid potential traits through the use of new precision phenotypic approaches and advanced high-density genomic tools (genome wide association studies and genomic prediction) [16,17,18,19,21,22,23]. Consequently, anther extrusion and profuse pollen shedding have been shown to be promising traits to predict related traits such as pollen mass, openness and duration of floral opening [16,19]. Similarly, selection of parents with the best parental trait combinations is a key determinant of the heterosis level [24]. However, potential male and female parents with superior floral and agronomic trait combinations may not always transmit desirable traits to their hybrid progenies. To overcome this issue, combining ability, as an important genetic parameter, has been widely adopted in plant breeding to compare the performance of lines in hybrid combinations [13,25,26,27,28].

The concepts of general combining ability (GCA) and specific combining ability (SCA) were first established by Sprague et al. [28]. GCA describes the average performance of a parent in different hybrid combinations, whereas SCA describes the deviation in performance of certain hybrid combinations as compared to what would be expected based on the GCA of the parents involved. GCA is an important indirect criterion in selecting inbred parents, which means that the phenotypic selection is based on the GCA [7], SCA might be used to identify a specific cross combination for exploitation through heterosis breeding [29].

A diallel analysis scheme was widely used to identify parental genotypes with high GCA and hybrid combinations with high SCA [30]; and to obtain the genetic information of hybrids and their parents for further classification in heterotic patterns [31]. To establish heterotic groups, selecting high-yielding parental lines with appropriate trait combinations could help in clustering the germplasm in different heterotic groups based on trait-per-se performance. Using this method, phenotypic and genotypic assessments of 196 genotypes for various floral and flowering traits were previously undertaken by El Hanafi et al. [19,32]. Our objectives in this study were to (1) investigate the efficiency of the CHA Croisor^®^ 100 on the selected female in crossing blocks using three different doses; (2) assess the hybrid seed set of successful hybrids produced using the appropriate rate; (3) investigate the genetic variance and heritability of the hybrid seed set and its correlation with the evaluated male floral traits; (4) evaluate the hybrid’s performance and obtain estimates of the expressed percentage of the mid-parent (MPH) and best-parent heterosis (BPH) levels; (5) and determine the patterns of GCA and SCA.

## 2. Results

### 2.1. Efficiency of the Chemical Hybridizing Agent

**Figure 1:**
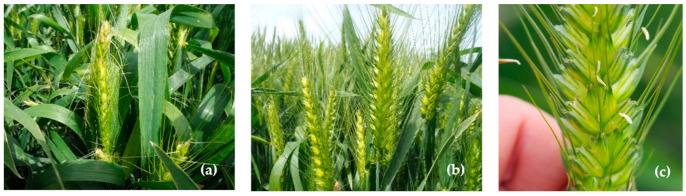
Reaction of the CHA on female plants; (**a**) phytotoxicity of the chemical; (**b**) gapping and light green color of a treated female; and (**c**) white-yellowish anthers.

The effectiveness of any CHA is environment and genotype dependent. Therefore, it was necessary to carry out tests in order to determine the appropriate rate for Moroccan conditions where spring bread-wheat is mainly grown. The application of the three different chemical doses 11.5, 12.5 and 13.5 L ha^−1^ based on the manufacturer’s recommendation were selected for their ability to induce sterility. The date of application on the wheat during the early booting stage (immature heads at 15 to 20 mm in the stem) differed based on environmental conditions each year. The staging was frequently and individually verified to meet the optimal time window for CHA treatment. Variation of the gapping date and different reactions to the chemical were observed. 12.5 L ha^−1^ dose was not sufficient to induce male sterility in the treated female lines (only a few sterilized plants produced seed) while 11.5 L ha^−1^ was not effective at all. However, almost complete male sterility and a slight phytotoxic effect were observed using the 13.5 L ha^−1^ rate (Figure 1a). Sterility control was verified by the number of seeds in the bagged female heads, sufficient gapping, a light green color observed as result of the effective dose, white-yellowish anthers that did not dehisce nor shed pollen, and the presence of waxiness on the leaf surfaces of the true F1 hybrids (Figure 1b,c). As result, we dropped the two unsuccessful rates (11.5 L ha^−1^ and 12.5 L ha^−1^) and we opted for 13.5 L ha^−1^ in the second-year experiment.

### 2.2. Relationship between Seed Set and Both Floral and Flowering Traits

Furthermore, we evaluated the flowering and floral traits of the males used and tested the hybrid seed set on the females. We observed large phenotypic variation for the assessed traits (Table 1 and Table S1). For the effective 13.5 L ha^−1^ dose, we recorded a range of 24.5 to 47.05 seeds per head. Significant genotypic and genotype-by-year interaction variances were observed for all traits. However, the genotype-by-year interaction variance was lower than the genotypic variance for all traits except for anther length. Heritabilities were medium to high and ranged between 0.54 for spikelets per spike and 0.94 for pollen mass (Table 1). The P1/P18 cross revealed the highest value of seed set per spike (47.05) and showed the highest visual anther extrusion and pollen mass with 9 and 39.58 mg, respectively (Table S1). Males contributing to the best performing parental combinations were selected for their highest values of visual anther extrusion, pollen shedding and pollen mass traits. Parents P1 (QAMAR-6) and P10 (SOMAMA-9/ICARDA-SRRL-2) were the two males that contributed to the best hybrids with high seed set. The best female contributors were P18 (VEE/PJN//2*KAUZ/3/SHUHA-4/FOW-2), P6 (SERI.1B//KAUZ/HEVO/3/AMAD/4/ATTILA//PSN/BOW/3/ATTILA/5/KAUZ’S’/SHUHA-15) and P2 (KAUZ//ALTAR 84/AOS/3/TNMU/MILAN/4/MILAN//PSN/BOW) and were among the parents having the highest number of spikelets per spike.

The phenotypic correlations among the evaluated traits ranged between 0.11 and 0.98 with the strongest correlation being between grain yield per spike and seed set (Table 2). Seed set, as an important determinant of successful hybrid production, was significantly correlated with visual anther extrusion, pollen mass, pollen shedding and anther length, 0.94, 0.97, 0.91 and 0.45, respectively. There were also high correlations between seed set and both spikelets per spike and plant height, 0.79 and 0.63, respectively. The assessed male floral traits showed significant correlations with each other, with the highest value observed being between pollen mass and pollen shedding (r = 0.96), as well as with some of the evaluated agronomic traits.

### 2.3. Hybrid Performance and Heterosis

To evaluate the performance of the F1 hybrids produced using CHA Croisor^®^ 100, the resulting seed from the crosses was planted in each of the following years. The germination rate was a good indicator that the progeny from the chemical hybridization was fully functional. We observed phenotypic variability for all the traits in each year and environment and across years and environments (Table 3 and Table S2); and the heritability ranged from low to high with maximum value observed for the yield in the 2019 trial in Sidi El Aydi. Correlation between the traits identified relationships between grain yield and the yield components for the overall data set. There was a high positive effect of biomass (r = 0.77), thousand kernel weight (r = 0.75) and number of productive tillers per plant (0.69) on grain yield, whereas there was a moderate positive effect of spikelets per spike on grain yield, which recorded a 0.55 coefficient of correlation (Table 4).

**Figure 2:**
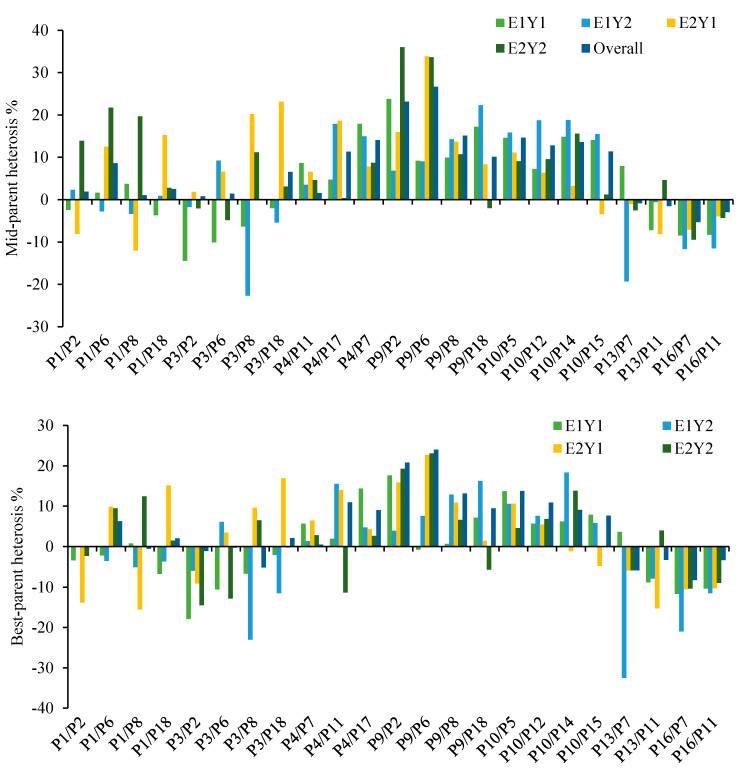
Mid- and best-parent heterosis for grain yield in Merchouch and Sidi El Aydi during the 2019 and 2020 seasons. E represents the environment and Y is the year; E1: Merchouch; E2: Sidi El Aydi; Y1: 2019; Y2: 2020.

All hybrids exhibited either positive or negative heterosis over the mid- (relative heterosis) and best- (heterobeltiosis) parent in each environment, and across environments (Figure 2, Figures S1 and S2). Since BM, TKW and YLD were the only significant traits in each year and across the years and environments, heterosis and combining abilities were discussed in detail for these three traits only.

Hybrid biomass ranged between 8.86–12.25 t ha^−1^, 9.08–13.43 t ha^−1^, 4.86–7.27 t ha^−1^, 4.61–8.23 t ha^−1^ and 7.14–9.48 t ha^−1^ in MER-2019, MER-2020, SEA-2019 and SEA-2020, respectively, and across years and environments. The majority of the hybrids falling in the 10 t ha^−1^ to 13.43 t ha^−1^ were in the Merchouch environment, with the highest value observed for the cross P9/P8 in the 2020 trial in Merchouch. The highest mid-parent heterosis (14.55%) for overall mean biomass (9.48 t ha^−1^) was recorded for the cross P10/P12 which performed very well in Merchouch and Sidi El Aydi during the two year experiment (Figure S1). The P10 parent (SOMAMA-9/ICARDA-SRRL-2) involved in three other crosses (P5, P14 and P15) significantly contributed to biomass performance and hence could be categorized as a high combiner. The two crosses P16/P7 and P16/P11 most often showed the least biomass in each year and in every environment as well as across years and environments (Table 3). Significant relative heterosis ranged from −7.64 to 14.55% for the combined data across years and environments with the highest heterosis recorded for the cross P10/P12. Compared with the single environments, maximum mid-parent heterosis was recorded in MER-2019, MER-2020, SEA-2019 and SEA-2020 with 27.88, 16.04, 24.95 and 50.24%, respectively. Best-parent heterosis ranged between −20.29–22.48% and −24.58–12.58% for the first and second year in Merchouch, respectively. While ranges −17.96–13.77% and −33.53–39.74% were recorded in the first and second year in Sidi El Aydi, respectively. Relatively low heterosis over the best-parent was calculated for the combined data with the lowest (−11.18%) and highest (7.21%) values for the two hybrid combinations P3/P8 and P13/P11, respectively.

Thousand kernel weight ranged between 33.44–42.14 g, 36.42–48.14 g, 26.51–40.10 g, 30.37–40.96 g and 32.79–41.19 g with the best performing hybrids recorded for P9/P18, P10/P14, P9/P6, P10/P15 and P9/P6 in MER-Y1, MER-Y2, SEA-Y1 and SEA-Y2 and across the four environments, respectively (Table 3). P9 (ANGI-2/HUBARA-3) and P10 (SOMAMA-9/ICARDA-SRRL-2) were shown to be good combiners and had high TKW values in and across environments (Table 3). For the two year’s data, the trials in Merchouch and Sidi El Aydi had a wide variation of mid- and best-parent heterosis, reaching up to 32.83% in the second-year trial in Sidi El Aydi (Figure S2). The best hybrid combinations showed consistently better mid- and best- parent heterosis in each and across environments and thus, MPH and BPH trends were almost similar. Hybrids with the P3 parental line (QADANFER-11/REBWAH-11) showed the lowest values for TKW for each year and across environments as compared with the rest of the crosses except for the hybrid P3/P6 in Merchouch for the second-year trial (Table 3). Similarly, the cross P16/P7, showing low biomass, also had lower TKW in all environments.

Hybrid grain yield in Merchouch in 2019 ranged from 3.84–5.26 t ha^−1^ with the highest yield observed for the cross between P10 and P5 (Table 3). In contrast, the 2020 trial in Merchouch showed more yield variation than the 2019 trial with maximum yield (6.12 t ha^−1^) recorded for the hybrid P10/P14. Among the 23 hybrids produced in Merchouch, 15 and 13 hybrids showed positive significant heterosis in the 2019 and 2020 trials, respectively. MPH in 2019 at Merchouch ranged from −14.42 to 23.76%, whereas in 2020, it ranged from −22.66 to 22.31% (Figure 2). At Merchouch, the crosses P9/P2 and P10/P14 showed the highest BPH for grain yield with 17.60 and 18.36% in 2019 and 2020, respectively. For the trials in Sidi El Aydi, the highest relative heterosis was shown by P9/P6 (33.86%) in 2019 and P9/P2 (36.02%) in 2020. Hybrids P10/P14 (18.36%) in 2019 and P9/P6 (22.71%) in 2020 exhibited the highest BPH in Sidi El Aydi. For the combined data, grain yield for the produced hybrids ranged between 3.81 and 5.26 t ha^−1^ with a maximum value expressed by P9/P6 (Table 3). Same-cross was identified as the most promising combination with the highest percent of mid- (26.65%) and best-parent heterosis (24.04%) (Figure 2).

### 2.4. Combining Ability

Combining ability analysis based on Griffing’s Method 3 was carried out for the 23 hybrids derived from the different parental combinations, and the variances are presented in Table 5. The genetic variance of the parental lines was considerably larger than the variance due to the GCA of the parents for all traits. Similarly, the environment variance for the parents analyzed separately was much higher than the variance due to GCA of the parents in combination with the environment for the evaluated traits. The error variances of the parental lines and hybrids were almost comparable. However, it is important to note that these estimates may have varied if we had a larger number of parental lines and therefore must be taken with caution. The analysis of variance also showed a significant effect on the GCA estimates of the evaluated traits except for SPS, SPL and TLP while the effects of the SCA were not significant except for yield. The ratio of GCA to SCA was greater than one for TLP, BM, TKW and YLD.

BLUPs values across environments calculated from the combining ability analysis provided an estimate for GCA values for each parental genotype (Table 6). Out of the 18 parents used, only a few had a GCA estimate significantly different from zero (*p* < 0.05) for each trait. The parents P10 (SOMAMA-9/ICARDA-SRRL-2) and P12 (ATTILA*2/PBW65//PFAU/MILAN) were identified as having high and significant GCA values for all traits except for TLP. Meanwhile, the two parents P5 (KAUZ//ALTAR 84/AOS 3/KAUZ/3/SHUHA-4//NS732/HER/4/QAFZAH-33) and P9 (ANGI-2/HUBARA-3) had significant GCA for BM, TKW and YLD. Hybrids involving these two potential parents performed well and showed high MPH and BPH. Parent P9 had the highest positive GCA score (560 kg ha^−1^) for grain yield while P16 (ATTILA//VEE#5/DOBUC’S’/3/QADANFER-9) had the poorest GCA score with a value of −540 kg ha^−1^ (Table 6).

The specific combining ability effects for the evaluated traits using the combined data set in each parental combination are presented in Table 6. From a total of twenty-three crosses, only five, P3/P2, P3/P8, P3/P8, P3/P18 and P10/P5, showed significance for SCA for plant height (*p* < 0.05). SCA effects for days-to-heading, spike length, spikelets per spike and tillers per plant were not significant. Meanwhile, P9/P2 and P10/P5 demonstrated significant positive SCA for biomass, with a maximum value of 1.24 for P9/P2. Unlike the above traits, fourteen cross combinations out of twenty-three showed significant negative or positive SCA values for thousand grain yield (*p* < 0.05). The highest SCA was estimated for the hybrid P9/P8 (+5.39), while the lowest value of SCA was obtained for the hybrid P3/P18 (−5.89). Similarly, most of the cross combinations with significant SCA effects for TKW were found to be present for yield as well. The crosses P9/P2 and P9/P6 ranked as the top two best combinations with +1.27 and +1.21 SCA values, respectively. In contrast, the hybrid P16/P7 showed the highest negative SCA effect for yield with −0.91. Taking all the cross combinations into consideration, P9 and P10 appeared to be the best parents since six out of seven crosses expressed significant positive SCA effects.

## 3. Discussion

Efforts to investigate the potential of hybrid breeding to further increase yield in bread wheat were carried out in this study. Understanding the genetic basis of the floral and flowering traits to enforce outcrossing ability may lead to an effective hybrid system and thus, achieve large-scale hybrid production. Successful hybrid wheat breeding programs rely on an optimized breeding strategy that combines several prerequisites for maximum effective pollination and seed set. Identification of parents with suitable hybrid traits, application of effective CHA with proper doses and timing, determination of the combining abilities and heterosis are key factors that determine the success of hybrid wheat breeding.

### 3.1. CHA Efficiency

In this study, we used parental lines identified previously by El Hanafi et al. [19] with favorable floral traits to assess the impact of male floral traits on female seed set, in designed crossing blocks. First, the efficacy of the gametocide Croisor^®^ 100 was tested using three different doses applied at early booting stage. This CHA is widely used particularly in Central Europe and has proven its commercial value for hybrid seed production. Frequent verifications were made for well-timed application. The sterility control mechanisms applied proved the efficiency of the chemical, and adequate gapping was observed. Male sterility was induced in almost 95% of the female plants using the 13.5 L ha^−1^ rate. Afterwards, seed data was collected to determine the success level of the CHA-treated female plants. The different parental combinations showed significant phenotypic variation of the hybrid seed set and other evaluated flowering and floral traits. The 13.5 L ha^−1^ dose plants had an average of 35 seeds on the mother lines which supplied enough seed for hybrid performance trials.

### 3.2. Variation in Cross-Pollination Traits

The significant genotypic variances recorded for the important floral traits such as pollen mass, visual anther extrusion, pollen mass and anther length suggest potential for exploiting the variation present in the genetic material used. Moreover, the high heritability recorded for all traits indicates that there is sufficient variation in the population used and there is low influence from environmental factors. This also suggests that the genotypes might be efficient for hybrid seed production in several environments. Similar variation and heritability were observed in previous studies confirming that these traits can be improved by consecutive hybridization for an efficient hybrid breeding program [16,17,19]. Anther extrusion, pollen mass and profuse pollen shedding were shown to be promising traits to act as proxies for predicting other harder to measure floral traits such as openness of the florets and duration of floral opening of female plants. However, little is known about the relationship with seed set and floral traits. Until now, Boeven et al. [17] was the only study that showed a correlation between visual anther extrusion and hybrid seed set (r = 0.76, *p* < 0.001). In this study, seed set was correlated with visual anther extrusion with r = 0.94 (*p* < 0.001). Thus, visual anther extrusion can serve as a rapid proxy to estimate the potential of a female line in a hybrid breeding program. The present study also addresses, for the first time as far as the authors know, the effect of other floral traits on seed setting. High correlations were found between seed setting and pollen mass, pollen shedding and anther length, with r = 0.97 (*p* < 0.001), r = 0.91 (*p* < 0.001) and r = 0.46 (*p* < 0.05), respectively. Surprisingly, the relationship between seed setting and the phenology traits, produced a non-significant correlation even though male lines were specifically chosen to flower 1–3 days earlier and to be taller than the female lines. This demonstrates the inaccuracy of using phenology traits to produce high seed set on female lines. Therefore, genomic tools might be incorporated to predict the flowering synchronization in hybrid seed production based on historical weather information [33].

Our study showed that pollen mass had a high correlation with pollen shedding (r = 0.96) and visual anther extrusion (r = 0.95; *p* < 0.001), and as was expected, the maximum extruded anthers contributed to maximum shed pollen outside the florets [34]. This confirms the results previously found by El Hanafi et al. [19] and Langer et al. [16]. Moreover, anther length can, in turn, increase the quantity of pollen released from every single anther which is confirmed by the collective correlation observed between pollen mass and each of anther length (r = 0.55), pollen shedding (r = 0.60) and visual anther extrusion (r = 0.55). Similar associations were reported by [16]. In contrast, there was no association between anther length and pollen mass or any other floral trait in a previous experiment carried out in Morocco [19].

### 3.3. Hybrid Performance and Heterosis

Successful hybrid seed was produced to test the 23 F1 hybrid combinations in yield trials across two cropping seasons in Merchouch and Sidi El Aydi. The genotypic variability for days-to-heading, plant height, spike length and tillers per plant were mostly found to be non-significant. The significant G × E observed for PLH, SPS, BM, TKW and YLD indicated that for at least some of the hybrid combinations, the cross and its parents, exhibited different levels of phenotypic expression under different environmental conditions. While for the rest of the evaluated traits (DH, SPL, TLP), G × E interaction effects were non-significant indicating the stability of the evaluated hybrids and their parents across the environments.

The main target of the hybrid breeding program was to identify potential parents that can be crossed to produce F1 hybrids with a high heterosis level. Previous studies have reported important heterosis and heterobeltiosis in wheat [13,35,36,37,38]. In this study, several hybrids exhibited significant heterosis and heterobeltiosis for the evaluated traits.

In this study, MPH for biomass ranged from −19.18 to 50.24% in the four environments. While a maximum of 13.35% was recorded for the overall data, expressed by the hybrid P9/P2. A maximum of 50.24% was recorded by the hybrid P1/P2 in the 2020 trial in Sidi El Aydi even though the parental pair involved had not shown the best biomass performance. This is not surprising considering that the cross performed better in extreme drought station (Sidi El Aydi) with supplemental irrigation as compared with a station in drought and only rainfed (Merchouch). However, negative mid- (−9.34%) and best-parent heterosis (−11.58%) was recorded for the same cross in the 2019 trial in Sidi El Aydi and attests to the instability of this cross across environments.

Stable positive heterosis over the mid-parent for biomass was recorded by six hybrids, P1/P8, P9/P2, P9/P18, P10/P5, P10/P12 and P13/P11, across each year and environment. This might be explained by the use of parental lines that were well characterized for potential floral traits combined with good agronomic performance. The wide positive range found for biomass heterosis was far better than what was found in previous studies with a maximum of 0.9% in Morgan et al. [39] and 5.4% in Kindred and Gooding [40]. BPH ranged from −33.53 to 39.74% in the four environments with a maximum value observed for the hybrid P10/P12 in the 2020 SEA trial. The maximum BPH for biomass for the combined data set was low (7.21%), displayed by the hybrid P13/P11.

For thousand kernel weight, a wide range of mid- and best-parent heterosis was found for the individual experiments and for the combined data. Some hybrids consistently outperformed their parents across all environments. Of the twenty-three hybrids produced, ten (P4/P17, P4/P7, P9/P2, P9/P6, P9/P8, P9/P18, P10/P5, P10/P12, P10/P14 and P10/P15) had consistently positive MPH in all environments except the cross P9/P18 in the 2020 SEA trial. This appears very promising especially because the parents were shown to have a dual parentage purpose (male and female). Many studies have reported positive and negative mid- and best-parent heterosis [41,42,43]. Singh et al. [42] reported the highest relative heterosis and heterobeltiosis of 28.14 and 24.07%, respectively, in three different environments. While two other studies reported a maximum of 16.14% MPH. Generally, greater MPH or BPH might be attributed to genetically distant parental lines that belong to different heterotic groups [44].

Grain yield heterosis in wheat has been a trait of widespread interest to many researchers from as early as 1934 [45]. Briggle [46] was among the first to report heterosis in wheat and since then research efforts over the years have demonstrated predictable yield advantage and stability [8,13,35,47,48,49,50,51,52,53]. Easterly et al. [35] evaluated 650 hybrids developed from a full diallel of 26 parents in experimental yield plots and reported mid- and best parent heterosis of a maximum of 24%. Adhikari et al. [13] reported ranges from −70.4 to 54.3% and −26.9 to 29.2% grown in two cropping seasons in Lincoln, NE, and Greenville, TX, USA. In the current study, MPH ranged from −14.41 to 23.76% and from −22.66 to 22.31 in the 2019 and 2020 Merchouch trials, respectively. While, in Sidi El Aydi, MPH tended to be better with ranges of −12.01 to 33.86% and −9.47 to 36.02% in 2019 and 2020, respectively. Hybrids have been shown to out-yield their best parents by 18.36% and 23.07% as seen with the highest values found at Merchouch and Sidi El Aydi, respectively. The higher positive MPH and BPH found in Sidi El Aydi might be attributed to a greater response to irrigation applied during the critical time of vegetation growth. For the combined data across locations, the cross P9/P6, considered as the best performing hybrid, was shown to out-yield the mid and best-parent by more than 1 t ha^−1^ each or 26.65% and 24.04% BPH, respectively. High yield of individual hybrids was observed in crosses involving high-yielding parents with promising parental floral traits as previously evaluated (P4, P9 and P10). In contrast, some other hybrids did not perform as expected even though both parents were reported to be high yielding, indicating that heterosis depends not only on performance per se but also combining ability.

Competitive hybrid wheat production depends on the heterosis level of yield to be sufficiently high to compensate for seed cost. It has been demonstrated in a previous study that cost-efficient hybrids would only be economically viable if the range of MPH was between 6 to 34% [54]. Similarly, Angus [55] estimated that 5% yield advantage over the best line-bred was required to counterbalance the higher seed cost. Thus, we can conclude that our results were very promising and the heterosis level demonstrated the potential of hybrid wheat to boost global wheat productivity and hence, show commercial hybrids to be viable.

### 3.4. General and Specific Combining Abilities

Several studies have estimated the GCA of each parental genotype and the SCA for each hybrid combination [8,13,28,35,41,52,53,56]. The analysis of combining ability provides an indication of the nature of gene action involved in the expression of the traits. The ratio σ^2^GCA/σ^2^SCA determines the preponderance of either additive or nonadditive type gene action. Higher σ^2^GCA to σ^2^SCA ratio was observed for TLP, BM, TKW, and YLD indicating the importance of additive gene action for the inheritance of these traits [57,58]. Reif et al. [59] reported that higher σ^2^GCA to σ^2^SCA ratio generally indicated the genetic dissimilarity of the parents used. Similar results were observed by Gowda et al. and Adhikari et al. [13,52]. Similarly, we completed a cluster analysis of the initial panel from which we selected the promising genotypes for hybrid production, which showed that the parents used in this study were mostly from different genetic clusters [19] (Figure S3).

In this study, the magnitude of the GCA effects was relatively higher in some of the parental lines for certain traits such as BM, TKW and YLD. Seven parents (P4, P5, P9, P10, P12, P14 and P17) had significant GCA effects for yield. Interestingly, of these seven identified parents, five (P5, P9, P10, P12 and P14) were identified as good combiners, having significant GCA effects for biomass and thousand kernel weight as well. It was evident that genotypes that showed high GCA effects for yield also demonstrated high GCA for some of the yield components such as biomass and thousand kernel weight. Similarly, we have shown these two traits had high direct effect on grain yield with 0.77 and 0.75, respectively. These results were in agreement with earlier findings [13,53]. Parental lines with high GCA effects may be used in future crossing strategies as improved parents for F1 hybrid production.

Significant positive SCA effects on biomass were observed in two crosses, P9/P2 and P10/P5 which both involved high × high general combiners as parents. These two crosses along with six others (P4/P7, P9/P6, P9/P8, P10/P12, P10/P14 and P10/P15) exhibited significant positive SCA effects on TKW and YLD. Of these eight hybrids exhibiting significant SCA effects in a desirable direction identified for TKW, four of them (P10/P5, P10/P12, P10/P14 and P10/P15) involved high × high general combiners as parents while four hybrids (P4/P7, P9/P2, P9/P6, and P9/P8) involved high × low general combiners. The negative SCA effects observed in some of the hybrids produced may have been due to the high and contrasting specialization of the two parents involved for yield formation or due to the presence of unfavorable gene combinations in the parents. The hybrids with high and positive SCA effects are recommended for hybrid breeding.

Because best specific combiners were not particularly derived from high × high general combiners as parents but also obtained from the combination of high × low general combiners, the high GCA effects of the parents were not necessarily reliable criterion to predict high SCA effects. Rewale et al. [60] have reported that the strong performance of the hybrids having high SCA might be attributed to additive × dominance gene action in the case of high × low GCA parental lines or to an epistatic interaction in the case of the parental pair having low GCA (a case that was not reported in this study).

## 4. Materials and Methods

### 4.1. Plant Materials and Field Experiments

A total of 18 elite bread wheat (*Triticum aestivum* L.) parents were selected from the ICARDA bread wheat breeding program based on their specific hybrid potential traits demonstrated by El Hanafi et al. [19], for their genetic dissimilarity (Figure S3), for their tolerance to drought and yellow rust resistance as reported by El Hanafi et al. [61] (Table S3). The male lines were taller than the females, with long extruded anthers producing good amounts of pollen, while the female lines were shorter with a wide and extended floral opening and prolonged stigma receptivity. The parents were all selected for grain yield and adaptation in different environments and their flowering nick was well synchronized. Seven male lines and eleven female testers were used for this study (Table S3). Experimental hybrids were made using Croisor^®^ 100 (active ingredient sintofen; 1-(4-chlorophenyl)-5-(2-methoxyethoxy)-4-oxo-1,4-dihydrocinnoline-3-carboxylic acid) (Asur Plant Breeding) sprayed on the female plots. We used three different rates 11.5, 12.5 and 13.5 L ha^−1^ to test the efficiency of the chemical. The chemical was sprayed according to the manufacturer’s recommendations, that is before flowering at stage 34 of the Zadoks scale [62] mixed with Heliosol at a rate of 0.3 mL m^−2^ as an adjuvant agent. The CHA performed as a male gametocide and the sterility in the female treated plots was verified by bagging individual spikes. CHAs are an easy and rapid hybrid production system that induce male sterility by a simple spray at the correct stage. Each crossing block consisted of different females surrounded by a unique male pollinator. To avoid pollen flow from any nearby plots, isolation walls were placed around every crossing block with early and tall barley (*Hordeum vulgare* L.).

**Figure 3:**
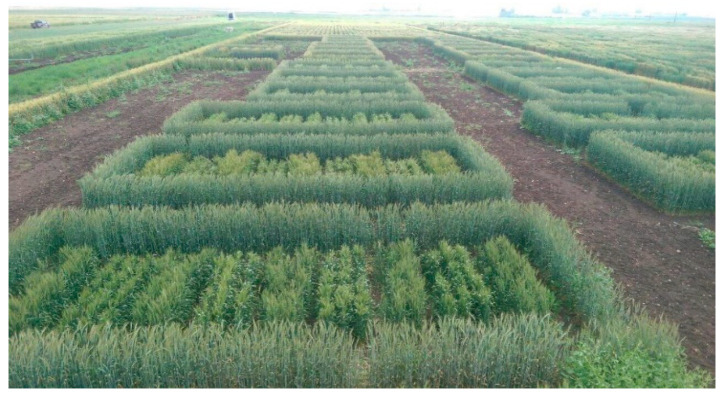
Experimental design to test the efficiency of the CHA used and female seed set.

To evaluate hybrid seed set and its relationship with other male floral and agronomic traits, the male lines were sown in two long strips of 5 m with 0.3 m spacing next to three female rows (Figure 3). Field experiments were conducted during 2017–2018 and 2018–2019 growing seasons at Merchouch Station, Morocco (33°36′ N, 6°43′ W, 394 m a.s.l.). All experiments were managed according to standard cultural practices. During the flowering stage, different traits were assessed following the same methods described by El Hanafi et al. [19]: (1) Visual anther extrusion (VAE) was visually scored for a plot based on a scale from 1 to 9 (1 = no anthers extruded, 9 = maximum anther extrusion). (2) Pollen mass (PM) in mg was recorded to estimate the released pollen from 10 randomly selected spikes placed into paper bags two days before anthesis. (3) Pollen shedding (PSH) was visually scored from five randomly selected spikes on a scale from 1 to 9 (1 = no pollen shedding and 9 = maximum pollen shedding). (4) Anther length (AL) in mm was recorded as the mean of nine anthers collected at flowering stage of nine different spikes using image analysis software ImageJ version 1.41 [63]. (5) Seed set was the average of seed count harvested from 10 randomly selected female heads. (6) Days to heading (DH) was recorded as number of days at 75% spike emergence in a plot (Zadoks GS 50) [62]. (7) Plant height in cm was measured from the ground to the spike tip without awns. (8) Spikelets per spike (SPS) was counted as mean spikelets per five random spikes of each plot. (9) Grain yield per spike in mg (GYS) was the mean weight of five random spikes of each plot. VAE, PM, PSH, AL, DH, and PLH were taken from the male plots at the flowering stage, while seed set, SPS and GYS were collected from female plots at post-flowering stage.

The hybrid seed produced from each production year (2018–2019) along with their parents were sown in two yield trials following an alpha lattice design with two replications in 3 m^2^ plots. The trials were conducted in a semi-arid region at Merchouch Station, Morocco (33°36′ N, 6°43′ W, 394 m a.s.l.) and in a dry arid location in Sidi El Aydi station, Morocco (33°12′ N, −7°62′ W, 236 m a.s.l.) during the 2018–2019 and 2019–2020 cropping seasons. Days to heading (DH) and maturity (DM) in Julian days, plant height (PLH), number of spikelets per spike (SPS), spike length (SPL), biomass (BM), thousand kernel weight (TKW) and grain yield (YLD) were recorded on a plot basis. SPS and SPL were collected accordingly on five randomly selected spikes for each plot.

### 4.2. Statistical Analyses

A linear mixed model using a restricted maximum likelihood (REML) method [64] was used to analyze the first data generated after the testcross using the chemical. Best linear unbiased estimates (BLUEs) were calculated for genotypes for all traits following the mixed model (1):

(1) ykmn = gk + lm + bnm + glkm + ekmn,

where ykmn is the phenotypic observation of the kth genotype in the nth block of the mth location, gk is the effect of the kth genotype, lm the effect of the mth location, bnm the effect of the nth block nested within the mth location, and ekmn is the residual plot error associated with ykmn. Genotypes were considered as fixed to obtain the estimated means in order to compute the correlations between the traits. Genotypes were considered as random to estimate the genotypic variance components and hence to compute heritability. Broad sense heritability (H^2^) of the evaluated traits was calculated from the expression (2) [65]:

(2) σ^2^g/(σ^2^g + σ^2^g·e/ne + σ^2^e/ne·nr),

where σ^2^g represented the genetic variance, σ^2^e the error variance, ne was the number of environments and nr the number of replicates. All models were fitted using ASReml v3.0-1 [66] in R v3.3.1.

To evaluate the produced F1 hybrids performance versus their parents, statistical analyses were performed on a yearly basis as well as across years and environments. A linear mixed model using a restricted maximum likelihood (REML) method was used for data analysis [64]. For yearly analyses, genotypes were considered as fixed whereas blocks were considered as random effects. BLUEs for each genotype were calculated for each genotype and each trait, and then used to compute traits correlations. Genotypes were taken as random to calculate the BLUPs and hence to get the genotypic variance components and then to compute heritability and the genotypic coefficient of variation. For the across years and environments analyses, years, environments and their interaction were taken as fixed effects and the genotype by year and environment interaction were random to obtain the genotypic and genotype by year interaction variance components. Broad sense heritability was computed following Equation (2).

Pearson correlation and its corresponding *p*-values were calculated between each trait, in each year. Narrow sense heritability (h^2^) was calculated from the expression [65] (3)

(3) σ^2^g/(σ^2^g + σ^2^g + σ^2^e/nr),

For each parent combination, mid-parent heterosis (MPH) (4) [67] and best-parent heterosis (BPH) (5) [68] for each location and environment as well as for the combined dataset were calculated as follows:

(4) MPH = (F1 − MP)/MP × 100%,

(5) BPH = (F1 − BP)/BP × 100%,

where F1 was the value of the hybrids produced; MP was the mean parent value; and BP represented the highest performing parent.

To evaluate the overall heterosis across locations and years, the BLUEs were used, while BLUPs were considered to estimate the combined GCA and SCA using the following model and using year and locations as replications.

(6) yijk = μ + gi + gj + sij + εijk,

where y was the phenotypic performance for the hybrid between the male and female genotypes i and j at kth environment, μ was the overall mean, gi and gj were the GCA effects of the ith and jth parents respectively, sij is the effect of the SCA for the combination ij and εijk was the residual random error for the observation in the kth block and with the parentals i and j. All variance components were determined by the restricted maximum likelihood method using the software ASReml-R version 3.0 [66].

## 5. Conclusions

Competitive hybrid breeding programs on a large scale require sustainable and robust hybridization systems. Development of hybrids through improved floral architecture has been shown to be valuable for maximizing seed set on female plants. The level of sterility induced in the female lines and the ability to produce higher seed set per head has demonstrated the success of CHA application and the beneficial utilization of wheat phenology. The heterosis found in this study appears to be enough to drive hybrid seed production and make it economically feasible. However, the genetic mechanisms of heterosis are not clearly understood. It is therefore necessary to dissect the genes involved in the regulation of heterosis. In such a way, the analysis of gene expression would lead to the genetic basis of the trait in question and heterosis would be conferred by either additive, dominant or epistatic action of the genes that control the trait. For instance, combining ability analysis also provides information on additive and non-additive variances which aids in selecting the desirable parents and crosses for the exploitation of heterosis. We have shown that the greater general combining ability estimates of the parents enables the prediction of the true genetic potential of an individual in subsequent segregating populations. Therefore, GCA is highly recommended to be performed in early generations in order to reduce time and cost in hybrid breeding programs. However, selection of hybrids based on high specific combining ability estimates alone would not prove effective since certain cross combinations may provide superior hybrids where some others involving equally promising parents subsequently produce poor progenies. Despite the fact that the combining ability method has many advantages, the use of genomic selection to identify single crosses may help in redesigning the hybrid breeding pipeline and thus shorten the time to hybrid release.

## Figures and Tables

**Table 1:** Estimation of genetic parameters for the evaluated traits at Merchouch station (2019–2020), Morocco.

Parameter	HD	PLH	SPS	GYS	PSH	VAE	PM	AL	Seed Set (Seed/Spike)
Min	99	96.5	18.3	1.336	6	6	26.545	3.5	24.5
Max	109	100	21.35	2.208	9	9	39.58	4.05	47.05
Mean	104.38	98.19	19.61	1.82	7.65	7.73	33.42	3.81	35.68
LSD	3.28	3.76	0.95	0.36	0.93	1.07	4.01	0.35	7.97
σ^2^_g_	6.27 **	10.63 **	2.22 ***	0.53 **	0.68 ***	1.04 ***	21.31 ***	0.63 *	19.23 **
σ^2^_g×y_	0.93 **	2.32 *	1.15 **	0.23 **	1.85 ***	0.36 ***	14.43 **	0.94 *	23.05 **
σ^2^e	0.43	0.47	1.20	0.17	0.27	0.36	4.84	0.53	69.51
H^2^	0.55	0.74	0.54	0.48	0.93	0.92	0.94	0.56	0.63

HD: heading date, PLH: plant height, SPS: spikelets per spike, GYS: grain yield per spike, PSH: pollen shedding, VAE: visual anther extrusion, PM: pollen mass, AL: anther length. Min, mean, max of BLUEs; least significant difference (LSD); σ^2^_g_ = genotypic variance; σ^2^_g×y_ = genotype-by-location interaction variance; σ^2^e = error variance; and broad sense heritability (H^2^). *, **, *** Significantly different from zero at the 0.05, 0.01 and 0.001 level of probability, respectively.

**Table 2:** Phenotypic correlations of seed set with other agronomic and male floral traits among the 18 parents of the combined data set across the two years.

Parameter	DH	PLH	SPS	GSP	PM	PSH	VAE	AL
PLH	−0.47 *							
SPS	0.11	0.63 **						
GSP	0.21	0.15	0.66 ***					
PM	0.28	0.25	0.81 ****	0.94 ****				
PSH	0.35	0.3	0.87 ****	0.86 ****	0.96 ****			
VAE	0.32	0.25	0.82 ****	0.90 ****	0.95 ****	0.95 ****		
AL	0.67 ***	−0.14	0.32	0.48 *	0.55 **	0.60 **	0.55 **	
Seed set	0.18	0.29	0.79 ****	0.98 ****	0.97 ****	0.91 ****	0.94 ****	0.46 *

*, **, ***, **** Significantly different from zero at the 0.05, 0.01, 0.001, and 0.0001 level of probability, respectively. Trait abbreviations are listed in Table 1.

**Table 3:** Performance of the parents and the hybrids; and the statistics summary for biomass, thousand kernel weight and grain yield, across years and environments.

Parents/F_1_ Hybrids	Biomass (t ha^−1^)					TKW (g)					Yield (t ha^−1^)				
	E1Y1	E1Y2	E2Y1	E2Y2	Overall	E1Y1	E1Y2	E2Y1	E2Y2	Overall	E1Y1	E1Y2	E2Y1	E2Y2	Overall
Parents															
P1	10.15	11.08	6.01	5.48	8.60	36.78	40.38	30.99	27.37	36.49	4.53	**5**	3.29	4.10	4.25
P2	9.83	9.78	5.71	4.27	7.42	37.35	**43.58**	30.36	25.73	34.1	4.44	4.77	3.76	3.08	4.08
P3	**12.37**	**12.21**	5.27	5.66	8.89	**40.6**	42.66	25.99	30.86	35	**4.84**	4.72	2.95	3.83	3.93
P4	9.78	10.72	5.13	4.47	7.77	35.19	40.67	28.65	30.51	34.71	4.3	4.74	3.37	3.59	4.26
P5	11.28	10.22	5.04	5.26	8.04	**38.22**	42.15	30.54	32.87	35.72	4.63	4.66	3.58	3.82	4.19
P6	**11.8**	8.99	5.25	4.67	7.65	**38.78**	37.38	34.14	28.03	34.7	**4.9**	4.72	3.14	3.44	4.07
P7	9.89	8.45	5.79	5.26	7.35	32.81	38.48	33.28	31.17	34.56	4.04	3.9	3.61	4.04	3.88
P8	**11.56**	10.63	5.17	5.39	8.22	36.43	40.7	33.45	29.73	34.83	4.81	**5.17**	3.58	3.78	**4.39**
P9	9	**12.06**	**6.39**	6.17	**8.95**	35.64	40.39	**39.66**	35.82	**38.77**	4	4.85	3.76	4.09	4.24
P10	10.21	**12.01**	6.24	5.59	**8.97**	36.85	40.71	**35.46**	34.34	**37.62**	4.56	4.73	3.61	4.16	4.25
P11	10.11	9.81	**6.34**	4.66	7.79	36.89	41.27	34.46	35.47	36.93	4.55	4.94	3.38	3.72	4.17
P12	10.55	8.58	5.10	5.89	7.58	37.12	36.79	**37.51**	**39.37**	**37.8**	4.7	4.17	3.67	3.95	4.11
P13	10.11	11.63	5.56	4.48	8.01	36.3	**46.01**	31.26	31.65	35.97	4.39	**5.09**	**4.01**	3.77	**4.32**
P14	9.45	11.61	5.76	5.98	8.12	33.42	42.76	34.25	32.95	35.95	3.87	4.87	**3.94**	4.03	3.92
P15	9.54	8.2	5.06	**7.48**	7.51	32.18	37.47	29.89	35.23	33.98	4.07	4.27	3.51	**4.25**	3.97
P16	10.09	10.48	5.26	**7.41**	8.32	36.41	**48.29**	31.95	31.82	36.99	4.35	4.24	**3.9**	4.12	4.14
P17	11.5	11.21	**6.27**	**6.94**	**9**	36.1	40.16	32.49	30.47	34.53	4.54	4.93	3.11	**4.39**	4.23
P18	11.56	10.87	5.14	6.26	8.5	37.15	40.44	31.97	**35.91**	36.42	**4.83**	4.54	3.28	**4.42**	**4.29**
F_1_ hybrids															
P1/P2	9.15	11.59	5.31	**7.33**	8.35	35.42	44.20	30.51	34.39	36.13	4.38	5.00	3.24	4.21	4.21
P1/P6	10.88	10.78	6.58	5.82	8.57	**41.30**	45.01	34.51	35.88	38.84	4.79	4.82	3.62	4.72	4.52
P1/P8	11.60	10.88	6.64	7.08	8.79	38.69	43.67	32.50	35.51	37.80	4.84	4.91	3.03	**4.85**	4.37
P1/P18	10.15	11.15	5.41	6.54	8.32	37.07	42.73	30.74	33.62	36.11	4.51	4.82	3.80	4.49	4.38
P3/P2	9.86	9.72	5.64	7.10	8.02	35.36	42.15	28.00	35.39	36.16	3.97	4.91	3.42	3.54	4.04
P3/P6	**12.22**	12.33	4.98	7.15	9.08	39.59	**46.44**	26.51	30.37	35.48	4.38	4.84	3.25	3.61	4.06
P3/P8	10.46	9.36	4.68	6.26	7.90	37.59	38.94	27.36	31.27	34.55	4.52	4.02	3.93	4.41	4.17
P3/P18	10.85	10.84	5.74	6.60	8.51	39.07	37.73	30.34	32.70	35.03	**4.74**	4.62	3.85	4.41	4.39
P4/P7	10.84	9.91	5.97	4.93	7.14	37.73	39.79	36.85	36.30	37.04	4.92	4.96	3.77	4.15	4.65
P4/P11	8.86	10.57	5.39	6.06	7.75	35.64	43.19	33.41	34.29	36.62	4.81	5.01	3.60	3.83	4.29
P4/P17	10.37	**12.86**	5.15	4.61	8.11	39.46	42.81	35.41	35.20	38.62	4.63	5.70	3.85	4.16	4.73
P9/P2	**12.04**	12.83	6.50	7.23	**9.28**	39.22	43.02	35.20	**40.88**	39.36	**5.23**	5.25	**4.37**	**4.88**	**5.13**
P9/P6	10.24	11.40	**7.27**	7.01	8.90	40.75	44.32	**40.10**	39.93	**41.19**	4.86	5.74	**4.63**	**5.04**	**5.27**
P9/P8	11.31	**13.43**	6.18	5.75	8.69	**40.79**	45.07	37.32	39.19	**40.59**	4.84	**5.84**	**4.18**	4.36	**4.98**
P9/P18	11.47	**12.95**	5.82	6.37	**9.13**	**42.14**	44.89	38.41	31.78	39.44	5.18	**5.87**	3.82	4.17	4.70
P10/P5	12.25	11.96	6.23	5.71	9.06	40.56	42.98	36.99	36.69	39.81	5.26	5.68	4.00	4.36	4.84
P10/P12	10.92	11.97	**6.78**	**8.23**	**9.48**	37.91	43.79	**39.00**	**40.36**	40.14	4.96	5.52	3.87	4.45	4.72
P10/P14	9.77	12.41	6.46	6.99	8.94	37.09	**48.14**	38.55	39.55	**40.78**	4.84	**6.12**	3.90	4.74	4.65
P10/P15	**10.77**	11.84	**6.90**	6.17	8.93	38.41	42.79	**39.45**	**40.96**	40.28	**4.92**	5.43	3.44	4.26	4.58
P13/P7	10.69	9.08	5.80	5.66	7.90	38.72	38.65	32.19	31.97	35.16	4.55	3.91	3.77	3.81	4.07
P13/P11	10.52	11.06	6.61	5.90	8.58	39.30	**47.01**	32.01	34.38	37.84	4.15	5.34	3.40	3.92	4.19
P16/P7	10.60	9.72	5.28	**7.25**	8.15	33.44	36.42	29.60	30.96	32.79	3.84	3.91	3.49	3.70	3.81
P16/P11	10.17	10.75	6.28	5.82	8.32	36.46	41.39	36.20	38.38	37.92	4.08	4.38	3.50	3.76	4.04
σ^2^_g_	0.13	0.25 *	0.39	0.53 *	12.25 *	9.62 *	10.6 *	3.51 *	8.92 *	2.63 *	0.21 *	0.07	0.16 *	0.18 *	0.82 **
σ^2^_L×E_	NA	NA	NA	NA	0.20 **	NA	NA	NA	NA	4.26 **	NA	NA	NA	NA	0.14 *
σ^2^_e_	1.28	0.59	1.90	0.72	1.20	5.77	12.81	9.01	12.74	9.76	0.37	0.15	0.25	0.13	0.26
h^2^	0.24	0.34	0.29	0.60	0.37	0.29	0.62	0.44	0.58	0.47	0.38	0.49	0.57	0.73	0.53

*, ** Significantly different from zero at the 0.05 and 0.01 level of probability, respectively. σ^2^_g_ genotypic variance, σ^2^_e_ error variance, σ^2^_L×E_ location × environment variance, h^2^ narrow sense heritability. E1: Merchouch location, E2: Sidi El Aydi, TKW: Thousand kernel weight. Bold values are the top three highest genotypes for that specific trait.

**Table 4:** Phenotypic correlations among traits in 23 F1 hybrids and their corresponding parents over the two year’s data.

Traits	DH	PLH	SPS	TLP	BM	TKW	YLD
PLH	0.83 ***						
SPL	−0.01	−0.18					
SPS	−0.2	0.07	0.12				
TLP	0.15	0.11	−0.07	0.41 **			
BM	0.29	0.18	−0.09	0.48 **	0.6 ***		
TKW	0.08	0.13	0.2	0.38 *	0.55 ***	0.56 ***	
YLD	0.03	0.06	−0.04	0.45 ***	0.49 ***	0.77 ***	0.75 ***

*, **, *** Significantly different from zero at the 0.05, 0.01, and 0.001 level of probability, respectively. DH: days to heading, PLH: plant height, SPL: spike length, SPS: spikelets per spike, TLP: tillers per plant, BM: biomass, TKW: thousand kernel weight, YLD: grain yield.

**Table 5:** Analysis of variance for the combining ability of traits for parents and their corresponding F1 hybrids.

Variance Component	DH	PLH	SPL	SPS	TLP	BM	TKW	YLD
Parents analyzed separately								
σ^2^_g_	5.45 *	4.40 *	0.60	12.54 *	0.91	0.30 *	0.22 *	0.16 *
σ^2^_L_	6.78	17.62	0.31	0.19	0.07	0.28	4.12	0.08
σ^2^e	1.38	12.88	60.84	91.29	90.22	1.95	11.23	0.93
H^2^	0.39	0.45	0.74	0.82	0.78	0.31	0.35	0.42
Hybrids analyzed separately								
σ^2^_GCA-parent_	0.19 *	2.64 *	0.03	0.07	0.05	0.08 *	0.09 *	0.07 *
σ^2^_SCA_	0	0	0	0	0.02	0.015 *	0.08 *	0.011 *
σ^2^_GCA-parent x L_	0.09	0	0	0.02	0	0.07	0.04	0.02
σ^2^e	3.31	11.45	53.61	101.17	82.46	2.91	9.27	1.06
σ^2^_GCA/_ σ^2^_SCA_	-	-	-	-	2.50	5.33	1.13	6.36
H^2^	0.29	0.52	0.31	0.72	0.69	0.48	0.53	0.68

* Significantly different from zero at the 0.05 level of probability. DH: days to heading; PLH: plant height; SPL: spike length; SPS: spikelets per spike; TLP: tillers per spike; BM: biomass; TKW: thousand kernel weight; YLD: grain yield; σ^2^g: genotypic variance; σ^2^L: location variance; σ^2^e error variance; H: heritability; σ^2^GCA: general combining ability variance; σ^2^SCA: specific combining ability variance; σ^2^GCA-parent × L: general combining ability combined with location variance.

**Table 6:** Estimates of GCA for the parental lines and SCA for the hybrids.

Parents/Hybrids	DH	PLH	SPL	SPS	TLP	BM	TKW	YLD
GCA Parents								
P1	5.45	4.40	0.60	12.54	0.91	0.30	0.22	0.16
P2	−0.85	−4.42 **	0.01	−0.38	0.17	0.01	−0.70	−0.05
P3	−0.74	−3.46 *	−0.08	−0.04	0.01	−0.13	−2.39 *	−0.30 *
P4	−0.87 *	1.53	−0.39	−0.32	−0.23	−0.85 *	−0.27	0.29 *
P5	−1.29	1.38	0.05	0.14	0.42 *	0.55 *	2.11 *	0.38 *
P6	−0.33	−0.24	−0.28	0.21	0.21	0.34	0.81	0.15
P7	−0.58	1.37	−0.19	−0.16	−0.26 *	−0.78 *	−2.70 *	−0.29
P8	−1.57 *	2.04 *	0.25	−0.23	−0.09	−0.05	−0.05	0.04
P9	−0.83	0.59	0.44	0.06	0.14	0.49 *	2.45 *	0.56 *
P10	1.51 **	2.83 *	−0.37 *	0.46 *	0.11	0.59 *	2.56 *	0.23 *
P11	0.58	−1.31	0.15	−0.32	−0.11	−0.30	−0.24	−0.29 *
P12	3.97 ***	5.04 **	−0.59 *	0.54 *	−0.07	0.97 *	2.44 *	0.36 *
P13	1.27 *	−1.78	−0.04	−0.11	0.10	−0.27	−1.20	−0.33 *
P14	1.01 *	3.35 *	−0.22	1.21 *	0.26	0.42 *	3.08 **	0.28 *
P15	2.35 **	1.54	−0.70 *	−0.05	−0.19	0.41	2.58 *	0.12
P16	0.07	−0.23	0.12	−0.19	−0.18	−0.28	−2.34 *	−0.54 *
P17	0.08	0.38	−0.86 *	−0.12	0.27 *	−0.41	0.92	0.27 *
P18	0.70	−1.35	0.86	0.31	−0.15	0.14	−0.84	0.03
SE	0.33	0.55	0.10	0.09	0.05	0.11	0.44	0.07
SCA cross combination								
P1/P2	1.14	2.94	0.51	−0.72	−0.29	−0.27	−2.17	−0.47
P1/P6	0.29	−2.50	−0.08	−0.10	0.01	−0.30	−0.30	−0.22
P1/P8	0.91	2.11	0.57	0.08	−0.14	0.30	−0.49	−0.26
P1/P18	0.02	0.59	0.19	0.09	−0.09	−0.36	−1.39	−0.23
P3/P2	−1.58	−7.50 *	−0.38	0.48	0.15	−0.64	−3.23 *	−0.67 *
P3/P6	1.85	−3.01	−0.19	0.09	−0.08	0.10	−5.41 *	−0.86 *
P3/P8	−3.29	−8.42 *	−0.53	−0.53	−0.14	−0.69	−5.49 *	−0.63 *
P3/P18	0.52	−7.47 *	1.37	0.37	−0.27	0.01	−5.89 *	−0.35
P4/P7	−1.53	4.00	−0.38	−0.17	−0.59	−1.44	1.77 *	0.57 *
P4/P11	−2.88	3.19	−0.65	−0.84	−0.47	−1.32	−1.11	0.21
P4/P17	−0.87	1.53	−0.39	−0.32	−0.23	−0.85	−0.27	0.09
P9/P2	−1.10	1.59	0.48	0.17	0.24	1.24 *	4.81 *	1.27 *
P9/P6	−3.67	2.54	0.88	−0.06	0.17	0.53	5.14 *	1.21 *
P9/P8	0.84	3.34	0.57	0.38	0.39	0.72	5.39 *	1.02 *
P9/P18	2.53	3.09	0.75	0.66	−0.44	0.12	−0.01	−0.27
P10/P5	1.08	8.64 *	−0.33	0.97	0.36	1.12 *	5.37 *	0.66 *
P10/P12	1.51	2.83	−0.37	0.46	0.11	0.59	2.56 *	0.23 *
P10/P14	1.51	2.83	−0.37	0.46	0.11	0.59	2.56 *	0.23 *
P10/P15	1.51	2.83	−0.37	0.46	0.11	0.59	2.56 *	0.23 *
P13/P7	2.59	−3.94	−0.19	−0.17	0.50	−0.10	−1.03	−0.44
P13/P11	2.49	−3.24	0.08	0.20	0.28	0.09	−0.82	−0.32
P16/P7	−0.58	−0.54	0.26	−0.28	−0.21	0.13	−4.55 *	−0.91 *
P16/P11	0.86	−0.43	0.26	0.01	−0.12	−0.18	−1.88	−0.67 *
SE	0.38	0.88	0.11	0.09	0.06	0.14	0.73	0.13

*, **, *** Significantly different from zero at the 0.05, 0.01, and 0.001 level of probability, respectively. DH: days to heading; PLH: plant height; SPL: spike length; SPS: spikelets per spike; TLP: tillers per spike; BM: biomass; TKW: thousand kernel weight; YLD: grain yield; GCA: general combining ability variance; SCA: specific combining ability variance SE: standard deviation.

## Data Availability

All the data, tables and figures generated are original.

## Supplementary Materials

The following are available online at https://www.mdpi.com/article/10.3390/plants11040508/s1, Table S1: Hybrid seed set using CHA at 13.5 L/ha. Table S2: Performance of the parents and the hybrids and the statistics summary for the traits evaluated across years and environments. Table S3: List of the 18 elite spring bread-wheat parental lines used in this study. Figure S1: Mid- and best-parent heterosis for biomass in Merchouch and Sidi El Aydi during the 2019 and 2020 seasons. Figure S2: Mid- and best-parent heterosis for thousand kernel weight in Merchouch and Sidi El Aydi during 2019 and 2020 seasons. Figure S3: Cluster analysis of genetic relationships among 196 genotypes used initialy to select the most promising lines for hybrid wheat production. The yellow marked lines were those used in this study. *, Genotypes with superior floral trait combinations.
