# Supplementary material for: Hybrid Seed Set in Relation with Male Floral Traits, Estimation of Heterosis and Combining Abilities for Yield and Its Components in Wheat (Triticum aestivum L.)
Source: Plants (Basel). 2022 Feb 14;11(4):508. doi: 10.3390/plants11040508 (PMC8880032; doi:10.3390/plants11040508)
Supplement: Supplementary file 1 [file plants-11-00508-s001.zip › Supporting Figures. Sel-Hanafi.pdf]

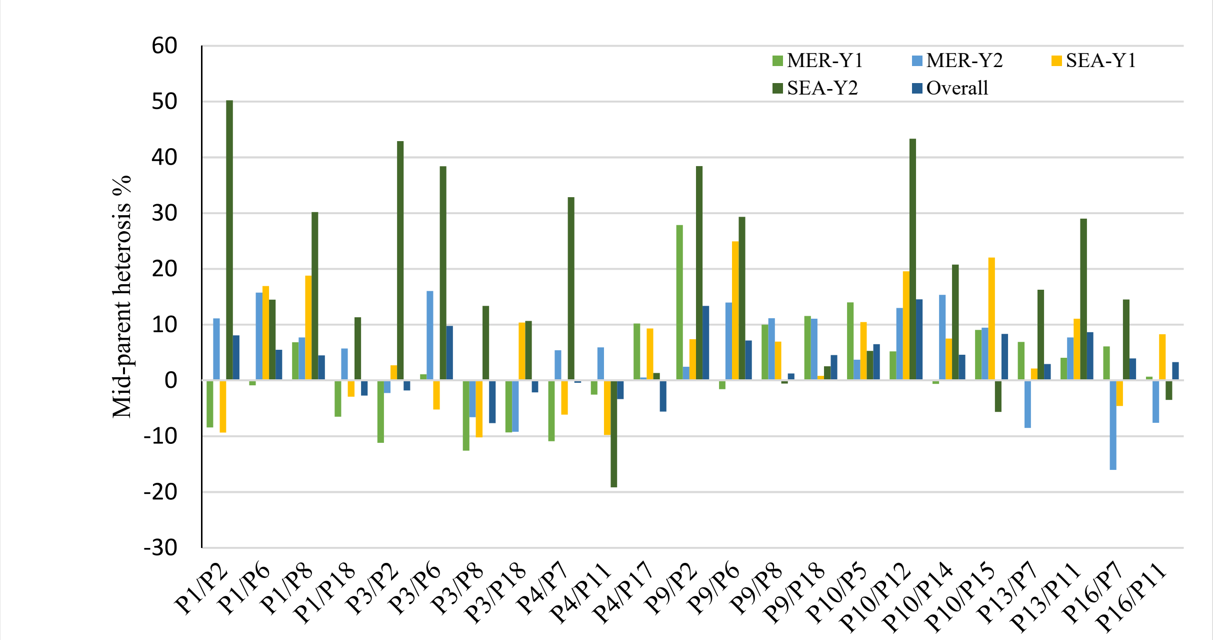

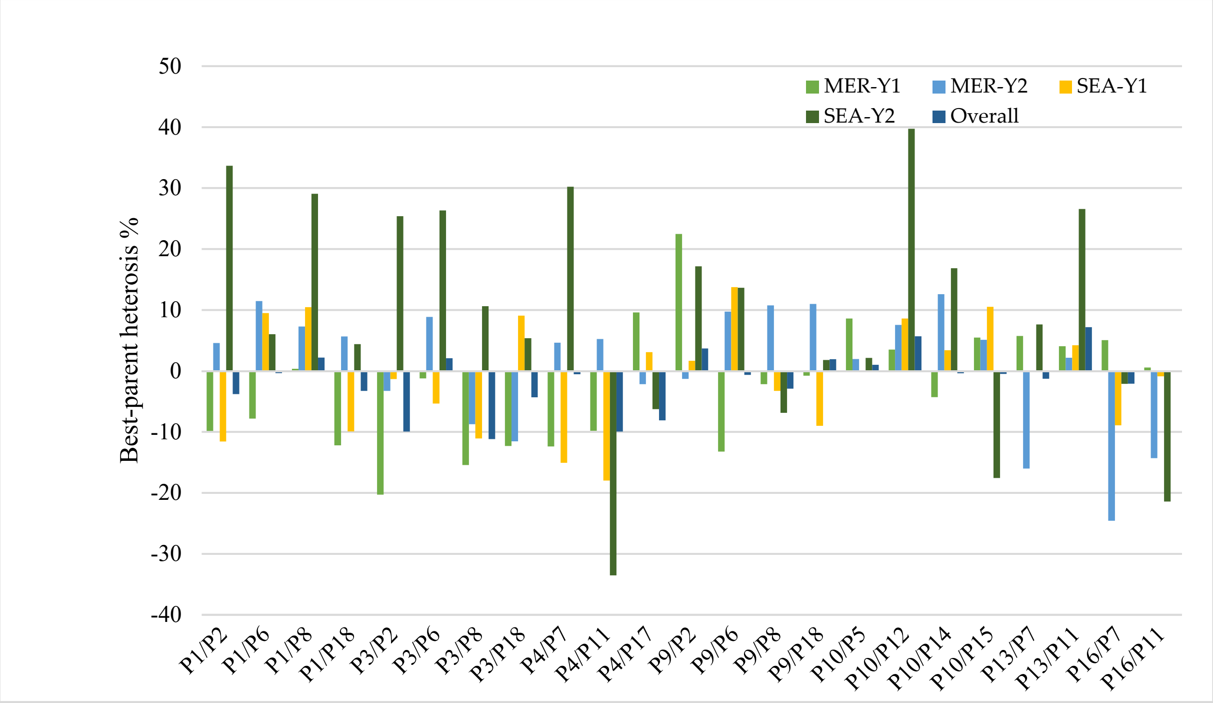

Figure S1. Mid- and best-parent heterosis for biomass in Merchouch and Sidi El Aydi during 2019 and 2020 seasons

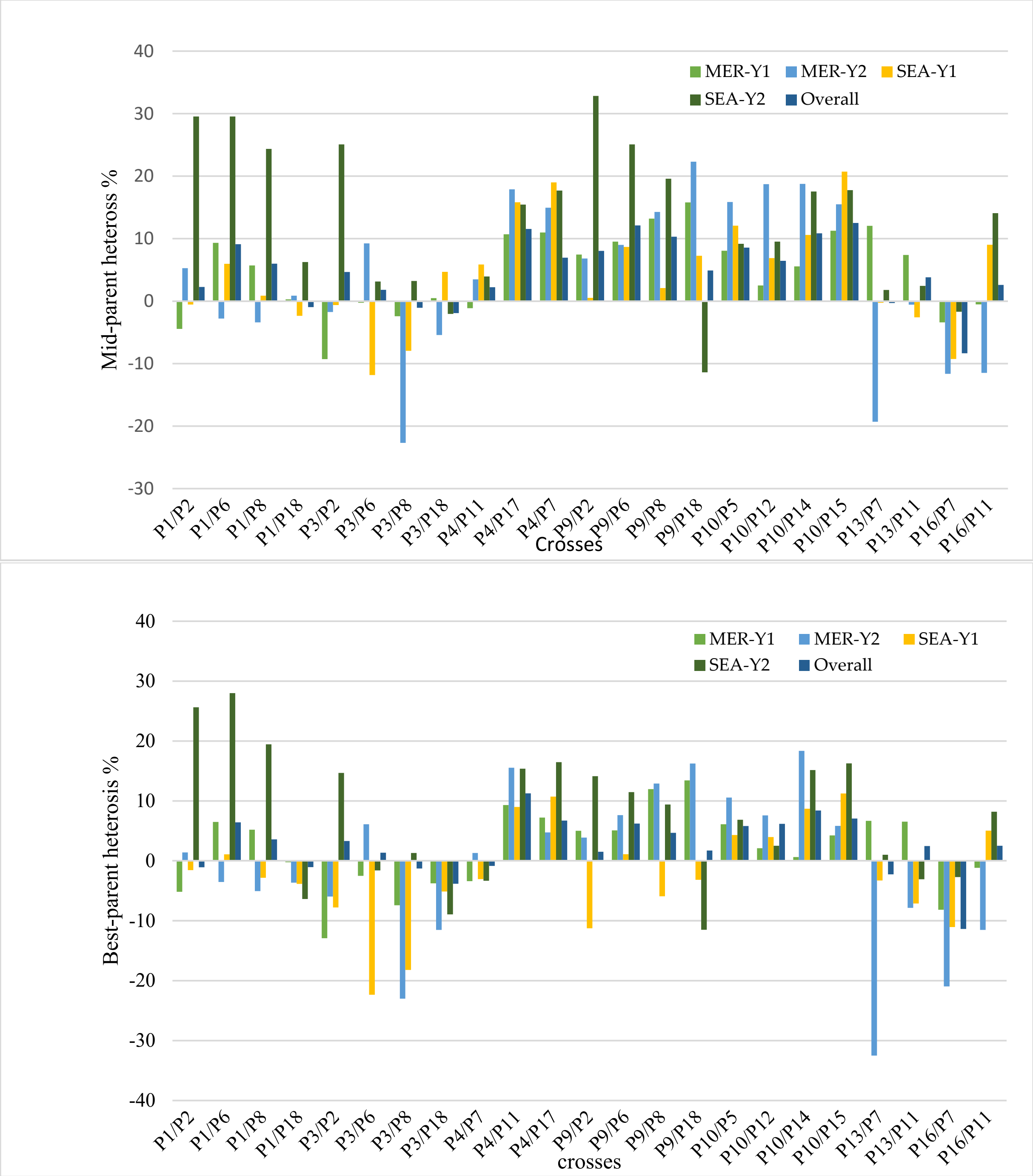

Figure S2. Mid- and best-parent heterosis for thousand kernel weight in Merchouch and Sidi El Aydi during 2019 and 2020 seasons

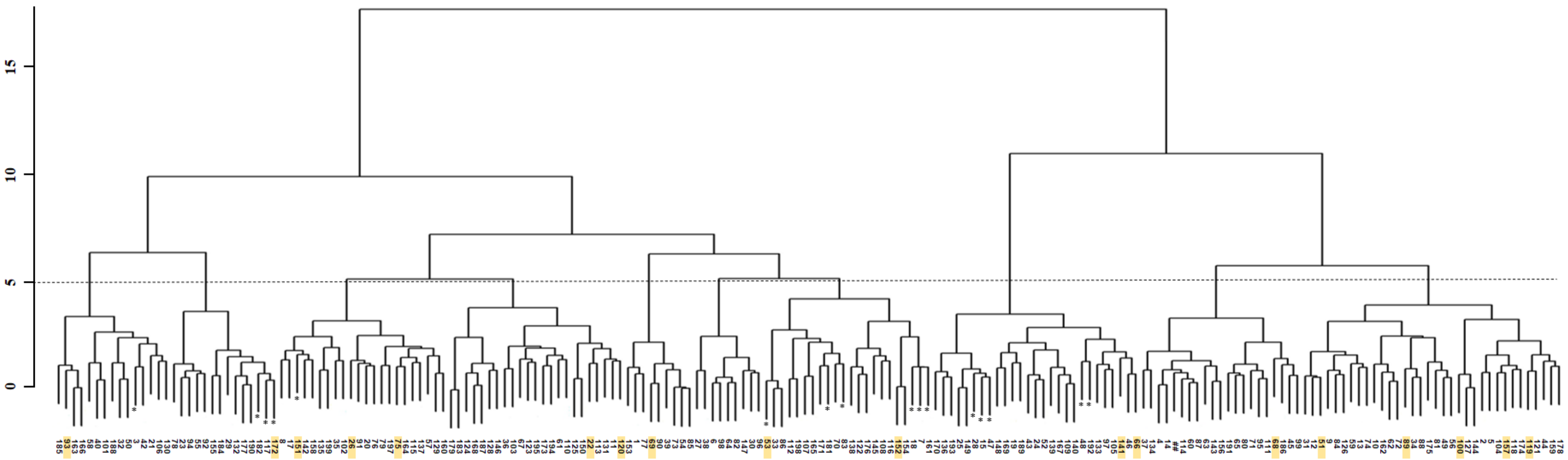

Figure S3. Cluster analysis of genetic relationships among 196 genotypes used initialy to select the most promising lines for hybrid wheat production. The yellow marked lines were those used in this study. *, Genotypes with superior floral trait combinations.
